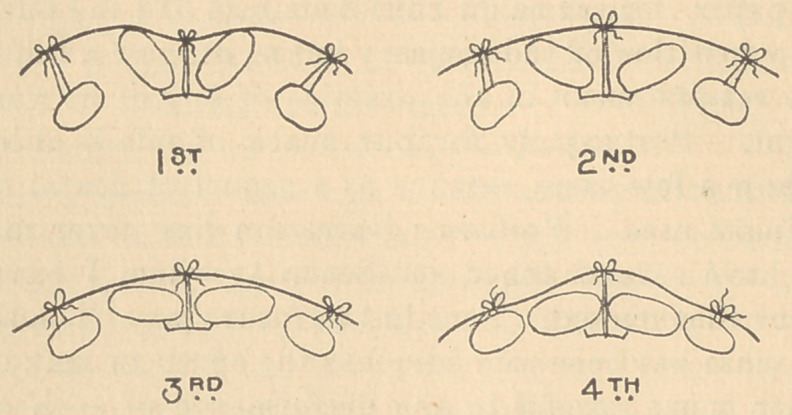# A Method of Correcting Irregularities

**Published:** 1891-07

**Authors:** J. Lehman Eisenbrey


### A METHOD OF CORRECTING IRREGULARITIES.[1]

BY J. LEHMAN EISENBREY, D.D.S.

WITH a narrow strip of gold plate—No. 25 or 26 United States gauge—and two thicknesses of gilling-twine, results most surprising can be gained in the way of correcting dental abnormalities. The gold strip must be lightly hammered to give it temper, or platinized gold may be used instead. With these we can draw, push, rotate, and crowd into position irregular teeth.

The case of Harry P., aged eleven to thirteen years, occupied about two years. I never push the operations, but prefer intervals of rest. And I find this method brings better, surer, and, strange as it may seem, quicker results.

To the question so often asked, When will the operation be completed? When finished we both will know it, so let us work earnestly in the present and let the matter take care of itself. If a time is fixed, and then not met, demoralization is the result. In these models before you, and the appurtenances, you see all that

1ST

2ND

3RD

4TH

was used in putting in position these ill-arranged teeth. The caps were worn for one year, day and night, being only taken off at meal-time or for cleansing. In the mean time the cuspids were coming through, and they were directed into position by daily pressure of the thumb long continued. The teeth became much discolored under the caps, but that was all removed by the felt-wheel and tooth-powder.

I have been asked why I do not use rubber tubing to help

[1] Read before the Pennsylvania Dental Society, July, 1890.

matters along? And my answer is, I do not want fast help, and, besides, it occasions intolerable and continuous pain. It allows of no resting period to the patients, and so breaks their little hearts.

The positive force of the gilling-twine—put on dry—exhausts itself in about two hours, and so the patient has courage to bear up for that length of time. The good work is still going on, though with bearable pain and soreness, in contradistinction to a pain that interferes with school studies.

The ligatures should be changed twice in each week.

After a tooth begins to move and absorption takes place, a very gentle pressure is all that is needed to keep up the absorptive action. Now, my reason for going slow is to give the reparative process a chance to take place, so as to hold all that is gained. Then, again, rapid movement means destructive inflammation, which is to be avoided, while up-building inflammation is necessary.

The case of Alice L., aged thirteen years, I present for your instruction and for the purpose of inciting discussion among you. Her lower maxilla is very prominent, with the molars only occluding. She is now wearing a draw-back bridle nightly, which will change the angle of the jaw. The upper maxilla I am expanding, and shall bring the circle forward a quarter of an inch. I will extract no teeth. The cuspid of the left side is, as you see, in the roof of the mouth. Next year I will cut it out.

The plates for expansion are movable, which is most desirable wherever possible.

The plate used for enlarging and drawing forward circle and rotating teeth is changed on Saturdays and Wednesdays and thoroughly disinfected. For this purpose there is nothing better than the imported phenol sodique, and for all or any condition of the mouth it is unexcelled. As a disguise, add some cologne. These models show five months' work, and another five will see them in their proper places.

These two patients were full of endurance, and may you all meet with like natures.

## Figures and Tables

**Figure f1:**